# Sleep and Trajectories of Respiratory and Allergic Symptoms Between 1 and 5.5 Years of Age in the Elfe Birth Cohort

**DOI:** 10.1111/jsr.70208

**Published:** 2025-09-16

**Authors:** Daniele Saade, Rosalie Delvert, Chantal Raherison‐Semjen, Orianne Dumas, Mohammed Sedki, Marie‐Noëlle Dufourg, Blandine de Lauzon‐Guillain, Bénédicte Leynaert, Rachel Nadif, Annabelle Bédard, Sabine Plancoulaine

**Affiliations:** ^1^ Université Paris Cité and Université Sorbonne Paris Nord, Inserm, INRAE Center for Research in Epidemiology and StatisticS (CRESS) Paris France; ^2^ Université Paris‐Saclay, UVSQ, Univ. Paris‐Sud, Inserm, Équipe d'Epidémiologie Respiratoire intégrative CESP Villejuif France; ^3^ Bordeaux University, Inserm, Bordeaux Population Health Research Center Team EPICENE, UMR 1219 Bordeaux France; ^4^ Service de Pneumologie Centre Hospitalier Universitaire Pointe‐a‐Pitre Abymes Pointe‐a‐Pitre Guadeloupe; ^5^ Université Paris‐Saclay, UVSQ, Univ. Paris‐Sud, Inserm Oncostat, CESP, Inserm Villejuif France; ^6^ Unité Mixte Inserm‐Ined‐EFS Elfe INED Paris France; ^7^ Université Claude Bernard Lyon 1, CNRS, INSERM Centre de Recherche en Neurosciences de Lyon CRNL U1028 UMR5292 Bron France

**Keywords:** allergy, birth cohort, children, clusters, longitudinal study, sleep

## Abstract

Sleep troubles and respiratory and allergic health issues are associated in children, but the timeline of their association is overlooked. This study investigates the associations between sleep patterns at age 1 and respiratory and allergic multi‐trajectories (RespA‐MTG) between ages 1 and 5.5, and the associations between these multi‐trajectories and sleep at age 5.5 in the ELFE birth cohort. Sleep clusters at ages 1 and 5.5 (based on nocturnal and diurnal sleep duration, sleep onset difficulties, and night awakenings) and RespA‐MTG between ages 1 and 5.5 (based on wheezing, asthma medication, eczema, allergic conjunctivitis) were identified using data‐driven methods. Associations between sleep clusters and RespA‐MTG were assessed using multinomial regressions adjusted for confounders in 9577 children. Two sleep clusters were identified at ages 1 and 5.5: good sleepers (79.9% at age 1, 83.1% at 5.5) and poor sleepers (20.1% and 16.9%, respectively). Four RespA‐MTG were identified: pauci‐symptomatic (44.4%), persistent non‐respiratory allergic symptoms (23.1%), transient early respiratory symptoms (25.2%), and persistent respiratory and allergic symptoms (7.3%). Poor sleep at age 1 was associated with higher odds of transient early respiratory symptoms (Odds Ratio [95% Confidence Interval], 1.14 [0.99–1.31]) and persistent respiratory and allergic symptoms (1.29 [1.05–1.59]). Results were reinforced in children without wheezing at 2 months. A borderline association was observed between persistent respiratory and allergic symptoms and sleep at 5.5 in good sleepers at 1 year (1.22 [0.98–1.50]). In conclusion, sleep disturbances at age 1 are associated with later poorer respiratory and allergic health, suggesting early sleep troubles may predict these health concerns.

## 1 Introduction

Asthma and sleep disruptions are two health problems experienced from childhood to adulthood in our society that have been both associated with subsequent poor health (Chaput et al. 2016, 2020; Ronco et al. 2022; Wee et al. 2021).

Asthma is a complex chronic inflammatory disease of the lower respiratory tract with multiple etiologies and variable clinical manifestations between individuals and over time (Stern et al. 2020). In 2019, the worldwide prevalence of ever‐asthma was 9.8% (Song et al. 2022), and asthma is the most common chronic disease in children (Asher et al. 2021; Delmas et al. 2023). About 25%–30% of children will have at least one episode of wheezing by age 6 years. In early childhood (preschool age), wheezing illnesses are common and consist of different phenotypes (e.g., transient or persistent wheezing). Recurrent/persistent wheezing is a strong predictor of asthma development (Asher et al. 2021; Dai et al. 2022; Delmas et al. 2023). Wheezing phenotypes are often associated with other allergic symptoms such as eczema, allergic rhinitis, and conjunctivitis (Anto et al. 2017; Apel et al. 2019; Bacharier and Guilbert 2019), also known to be associated with impaired quality of life and representing a substantial health burden (Callander and Schofield 2015; Carroll et al. 2005).

Sleep is vitally important to the health and well‐being of children and adults (Hirshkowitz et al. 2015; Paruthi et al. 2016). Healthy sleep requires adequate duration, good quality, regularity, and absence of sleep disturbances (Paruthi et al. 2016). However, sleep disturbances such as short night‐ and day‐sleep durations, frequent sleep onset difficulties, and frequent night waking affect 20%–30% of children during the first 3 years of life (Al Mamun et al. 2012; Bruni et al. 2014; Messayke et al. 2020). Previous studies showed that sleep troubles in childhood were tracked in adolescence and adulthood (Al Mamun et al. 2012; Sivertsen et al. 2017) and were associated with several negative mid‐ and long‐term consequences, including behavioural and cognitive performance and cardiometabolic troubles (Chaput et al. 2016; Quist et al. 2016; Reynaud et al. 2018).

Sleep disruptions, asthma and allergic symptoms are linked. Recent reviews report positive cross‐sectional associations between sleep disturbance (short night sleep duration, sleep onset difficulties, night waking) and allergic rhinitis (Liu et al. 2020; Schuler Iv and Montejo 2021) and atopic dermatitis/eczema in adults and children (Guo et al. 2023; Lee et al. 2023), and respiratory symptoms seen in asthma were associated with sleep deprivation and poor sleep quality in adults and children (Hu et al. 2020; Koinis‐Mitchell et al. 2012). Respiratory symptoms occurring during the night in asthmatic patients are usually considered a lack of control or a marker of severity of asthmatic disease (Krouse et al. 2008; Meltzer and Pugliese 2017), and well‐controlled or mild asthmatic patients (adults and children) are less likely to experience sleep disturbance (Krouse et al. 2008; Meltzer and Pugliese 2017). In addition, some longitudinal studies have shown a positive association between insomnia or poor sleep and incident asthma in adults (Brumpton et al. 2017; Xiang et al. 2023). The complex relationships between sleep and asthma suggest potential bidirectional effects (Kavanagh et al. 2018). However, a recent bidirectional Mendelian randomization study reported that adult insomnia may be a causal factor in adult allergic disease and asthma, whereas the reverse was not found (Li et al. 2022). In any case, sleep structure and day/night distribution, as well as asthma symptoms undergo important changes during childhood (Bathory and Tomopoulos 2017; Ranciere et al. 2013), and findings on the relationship between sleep and asthma in adults may not be transposable to children.

For a better understanding of the interrelations between sleep and respiratory and allergic health in preschoolers, this study aimed at (1) identifying clusters of sleep characteristics at 1 and 5.5 years old; (2) identifying longitudinal respiratory and allergic symptoms multi‐trajectories groups (RespA‐MTG) between ages 1 and 5.5 years; and (3) studying the longitudinal associations between sleep clusters at age 1 and 5.5 years and RespA‐MTG in the ‘Etude Longitudinale Française depuis l'Enfance’ (ELFE) birth cohort.

## 2 Methods

### 2.1 Study Design and Population

The ELFE birth‐cohort is a national cohort launched in 2011 in metropolitan France. Profile and design of this cohort have been previously published (Charles et al. 2020). In brief, 320 randomly selected maternity units participated. Inclusion criteria were infants born after 33 weeks of gestation to mothers ≥ 18 years old who agreed to participate, and a total of 18,329 infants were recruited. For the present study, withdrawn consent (*n* = 58), children for whom it was not possible to validate all inclusion criteria (*n* = 334), multiple pregnancies (*n* = 556), and children born preterm (< 37 gestational weeks, *n* = 758) were excluded, resulting in a study sample of 16,629 children. Based on data availability, 13,064, 10,504, and 10,524 children were included in the sleep clusters' identification at age 1 year and 5.5 years, and in the RespA‐MTG modelling, respectively. A total of 9577 children had clusters (at ages 1 and 5.5 years) and RespA‐MTG assignments and were included in the association analyses (Appendix S1).

ELFE study obtained approval from the Consultative Committee for the Treatment of Information for Health Research (CCTIRS), the national data protection authority (CNIL) and the National Council of Statistical Information (CNIS). Participating mothers signed a written consent for themselves and their infant. Fathers signed a consent for the infant's participation when present on inclusion days or were informed about their rights to oppose.

### 2.2 Data Collection

Face‐to‐face questionnaires, medical records at the maternity wards, and parental phone call interviews during the follow‐up phases at 2 months, 1, 2, 3.5 and 5.5 years were used to collect data. Questions used at each age to collect sleep, respiratory, and allergic symptoms data are presented in Appendix S2.

#### 2.2.1 Sleep Characteristics

At 1 year of age, night and day sleep durations were collected in hours and minutes. Frequent night waking and frequent sleep onset difficulties were recoded as present when they occurred ‘3 to 6 nights a week’ and ‘always’ for night waking and ‘often’ and ‘always’ for sleep onset difficulties. At the age of 5.5 years, average night sleep duration per day over the week was calculated in hours and minutes. Frequent night waking and frequent sleep onset difficulties were recoded as at 1 year of age. At each age, night and day sleep durations were categorised into tertiles.

#### 2.2.2 Respiratory and Allergic Symptoms Between 1 and 5.5 Years

Presence of respiratory and allergic symptoms (i.e., wheezing, asthma medication, eczema, allergic conjunctivitis) were collected in yes/no questions. Asthma medication was recoded as yes when the child received at least one inhaled bronchodilator or one inhaled corticosteroid and as no in all other cases. Allergic rhinitis was only collected at age 5.5 and thus was not included in the longitudinal modelling.

#### 2.2.3 Confounding Factors

Sociodemographic and maternal characteristics were collected via the medical records at the maternity ward and the 2‐month phone interview. Data collected were on maternal birth place, monthly household income in euros per consumption unit, maternal educational level, parity, maternal pre‐pregnancy body mass index (BMI), maternal age at delivery, maternal smoking during pregnancy, and maternal depressive symptoms during pregnancy, and maternal history of asthma.

Child's characteristics were collected from medical records and parental phone interviews. They included sex, gestational age, any breastfeeding duration, passive smoking exposure between 2 months and 1 year old, and main day‐care arrangement at age 1 year.

### 2.3 Statistical Analysis

#### 2.3.1 Descriptive Analysis

All analyses were performed using SAS version 9.4 (SAS Institute Inc., Cary, NC, USA). Characteristics of the study included and excluded children were compared using the chi‐square and *t*‐test to compare categorical and continuous variables, respectively.

#### 2.3.2 Identification of Sleep Clusters at 1 and 5.5 Years Old

Sleep clusters were identified separately at 1 and 5.5 years using latent class analysis (LCA) (PROC LCA procedure), an unsupervised data‐driven clustering method developed by Lanza et al. (2007). It detects latent (or unobserved) heterogeneity in samples, useful for identifying qualitatively different subgroups within a population who share certain outward characteristics. The number of clusters was determined based on the minimum Bayesian Information Criterion (BIC) and clinical interpretability. The child was assigned to the class or cluster for which he/she had the highest probability of belonging.

Sleep clusters at each age were identified among children with at least one available data point on sleep characteristics, including night and day sleep durations, frequent night waking, and frequent sleep onset difficulties at age 1 year, and night sleep duration, frequent night waking, and frequent sleep onset difficulties at age 5.5 years old (Appendix S1). Thus, sleep clusters were identified in 13,064 children aged 1 and 10,504 children aged 5.5 years. Complete sleep data was available for 98% and 97.3% of children at age 1 and 5.5 years, respectively.

#### 2.3.3 Identification of Respiratory and Allergic Multi‐Trajectories Between 1 and 5.5 Years Old

Respiratory and allergic symptoms multi‐trajectories (RespA‐MTG) were identified among 10,524 children having data available at least at 2 collection time‐points out of 4 possible between 1 and 5.5 years for all respiratory and allergic symptoms (wheezing, asthma medication, eczema, allergic conjunctivitis). The RespA‐MTG were identified using the group‐based multi‐trajectory modelling method (PROC TRAJ procedure) developed by Nagin et al. (2018) It allows both simultaneous modelling of multiple measures of the same underlying phenomenon and handling missing values. Different models with 1–5 groups were computed and compared based on the Bayesian Information Criteria (BIC). The final chosen model favours parsimony and its quality was verified according to the recommended criteria: the average probability of belonging to each group (≥ 0.7), the odds of correct classification (≥ 5), and the similarity between the model's estimation of the multi‐trajectory groups estimated and observed prevalence (Nagin et al. 2018). Children were then assigned to the multi‐trajectory group for which they had the highest probability of belonging.

#### 2.3.4 Association Analyses

Associations between sleep patterns at age 1 year and RespA‐MTG between 1 and 5.5 years and between RespA‐MTG between 1 and 5.5 years and sleep patterns at age 5.5 years were estimated by unadjusted and adjusted multinomial logistic regression models in children with available data for both sleep clusters at each age and RespA‐MTG (*N* = 9577). Covariates included in the adjusted models and described previously were the potential confounders identified from literature and selected using the Directed Acyclic Graphs (DAG) method (Tennant et al. 2021) (Appendix S3). To account for potential tracking of early respiratory and sleep troubles, regression models assessing the associations between sleep clusters at age 1 year and RespA‐MTG and between RespA‐MTG and sleep clusters at age 5.5 years were re‐run in children without wheezing at 2 months of age (*N* = 9054) and good sleepers at the age 1 year (*N* = 7814), respectively. A sensitivity analysis additionally included maternal history of asthma.

Multiple imputations of missing data for covariates (1.1%) were performed by using Fully Conditional Specification (SAS 9.4: MI procedure, FCS option). Binary variables were imputed by logistic regression, nominal and ordinal variables were imputed by multinomial logistic regression, and continuous variables were imputed by linear regression. Five databases were imputed. Estimates and confidence intervals were pooled to obtain overall results (SAS 9.4: MIANALYSE procedure) according to Rubin's rules (Rubin 1987). A *p* < 0.05 was considered significant for all analyses.

## 3 Results

### 3.1 Characteristics of the Study Population

Maternal and children characteristics for the excluded and included populations are provided in Appendix S4. Briefly, children included in the association analysis (*N* = 9577) were significantly more likely to be born in families with higher income, more educated mothers, primiparous and older mothers, but healthier and with less risky behaviours during pregnancy (lower pre‐pregnancy BMI, fewer depressive symptoms, less smoking) than excluded children (*n* = 7046). Also, included children had higher gestational age, longer breastfeeding duration, and less passive smoking exposure than excluded children. Child sex did not differ by inclusion. Descriptions of those characteristics in the other samples used either to identify sleep clusters or respiratory health and allergic multi‐trajectory groups are provided in Table 1.

**TABLE 1:** Children characteristics among the included children in the sleep clustering analyses at ages 1 and 5.5 years, the multi‐trajectories groups modelling and the final association analyses.

	Sleep clustering		Multi‐trajectories modelling	Final sample
	1 year old	5.5 years old		
	*N* = 13,064	*N* = 10,504	*N* = 10,524	*N* = 9577
	% (*n*) or mean (SD)	% (*n*) or mean (SD)	% (*n*) or mean (SD)	% (*n*) or mean (SD)
Socio‐demographic characteristics				
Maternal educational level				
< High school	37.7 (4929)	32.1 (3366)	30.4 (3198)	30.0 (2869)
High school	22.6 (2952)	23.7 (2479)	24.3 (2551)	24.1 (2312)
> High school	39.7 (5181)	44.2 (4628)	45.3 (4761)	45.9 (4396)
Household income (€/month/CU)				
≤ 1000	17.1 (2101)	13.8 (1385)	12.8 (1296)	12.5 (1154)
[1000–1385]	19.4 (2386)	18.4 (1848)	18.1 (1833)	18.2 (1676)
[1385–1662]	17.3 (2123)	17.8 (1787)	18.3 (1850)	18.1 (1665)
[1662–2078]	24.1 (2961)	25.9 (2597)	26.2 (2648)	26.4 (2436)
> 2078	22.1 (2708)	24.1 (2412)	24.5 (2473)	24.8 (2285)
Maternal characteristics				
Age (years)	31.0 (4.8)	31.3 (4.6)	31.5 (4.5)	31.4 (4.5)
Born abroad	11.0 (1440)	9.7 (1016)	9.0 (952)	8.8 (844)
Smoking during pregnancy	18.2 (2353)	16.4 (1711)	15.9 (1661)	15.7 (1496)
BMI before pregnancy (kg/m^2^)	23.4 (4.7)	23.3 (4.6)	23.3 (4.6)	23.3 (4.5)
Multiparous	66.4 (8647)	65.9 (6901)	65.5 (6865)	65.6 (6264)
Depressive symptoms during pregnancy	12.0 (1554)	11.9 (1237)	12.2 (1271)	11.8 (1119)
Children characteristics and early factors at 1‐year‐old				
Sex (boy)	49.1 (6408)	51.0 (5352)	51.0 (5368)	50.9 (4879)
Gestational age (weeks)	39.4 (1.1)	39.5 (1.1)	39.3 (1.4)	39.5 (1.1)
Breastfeeding duration (months)	3.8 (5.4)	4.0 (5.7)	4.0 (5.7)	4.1 (5.7)
Passive smoking exposure	4.2 (532)	3.8 (381)	3.5 (359)	3.5 (328)
Main day‐care arrangement				
Cared by family members	41.5 (5377)	36.8 (3727)	36.1 (3677)	35.7 (3392)
Collective care	16.1 (2087)	17.4 (1764)	17.5 (1784)	17.6 (1668)
Cared by employed person	42.3 (5478)	45.8 (4647)	46.4 (4734)	46.7 (4439)

Abbreviations: BMI, Body mass index; CU, Consumption unit; SD, Standard deviation.

### 3.2 Sleep Characteristics and Clusters

Among the children included in the sleep clusters identification, at age one year (*N* = 13,064), the mean night sleep duration was 10 h 43 ± 1 h 15, (tertile thresholds ≤ 10 h and > 11 h), the mean day sleep duration was 2 h 57 ± 0 h 59, (tertile thresholds ≤ 2 h 30 and > 3 h), in accordance with sleep duration recommendations for the age (11–14 h/24 h; Paruthi et al. 2016). The prevalence of frequent sleep onset difficulties and frequent night waking was 14.6% and 21.4%, respectively. At age 5.5 years (*N* = 10,504), the mean night sleep duration was 10 h 46 ± 0 h 31, (tertile thresholds ≤ 10 h 30 and > 11 h), also in accordance with sleep duration recommendations for the age (10 to 13 h/24 h, (Paruthi et al. 2016)). The prevalence of frequent sleep onset difficulties and frequent night waking was 12.1% and 6.7%, respectively.

**FIGURE 1:**
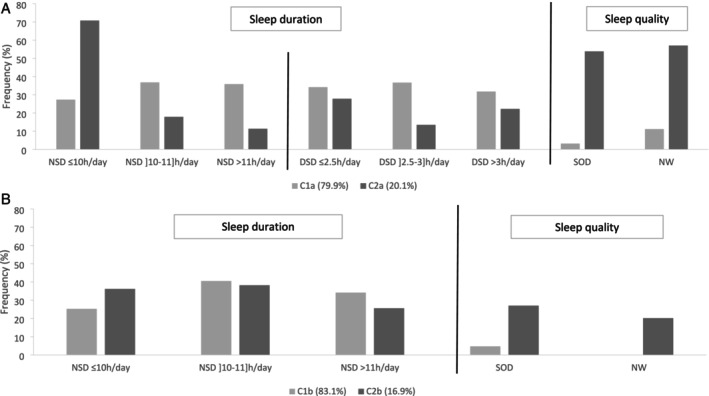
Clusters of children's sleep characteristics. (A) At age 1 year (*N* = 13,064). (B) At age 5.5 years (*n* = 10,504). DSD, day sleep duration; NSD, night sleep duration; NW, night wakening; SOD, sleep onset difficulties.

Two clusters were identified for sleep characteristics at age 1 year: C1a or good sleepers (79.9% of the children) with long night sleep duration (mean 10 h 59 ± 6 min), long day sleep duration (mean 3 h 00 ± 0.5 min) and no or few ‘frequent sleep onset difficulties’ and ‘frequent night waking’ (< 10%); and C2a or poor sleepers (20.1% of the children) with short night sleep duration (mean 9 h 37 ± 2 min), short day sleep duration (mean 2 h 42 ± 1 min) with frequent ‘frequent sleep onset difficulties’ and ‘frequent night waking’ (> 50%) (Figure 1A).

Two clusters were also identified for sleep characteristics at age 5.5 years: C1b or good sleepers (83.1% of the children) with long night sleep duration (mean 10 h46 ± 0.3 min) and no or few ‘frequent sleep onset difficulties’ and ‘frequent night waking’ (< 5%); C2b or poor sleepers (16.9% of the children) with slightly shorter night sleep duration (mean 10 h40 ± 0.5 min) and frequent ‘frequent sleep onset difficulties’ and ‘frequent night waking’ (> 20%) (Figure 1B).

A total of 10,196 children have information available for sleep clusters at age 1 year and at age 5.5 years. Among them, 27.7% of children in the poor sleeper cluster at age 1 were still within the poor sleeper cluster at age 5.5, and 14.3% of children in the good sleeper cluster at age 1 year became poor sleepers at age 5.5 years.

### 3.3 Respiratory and Allergic Health Between 1 and 5.5 Years

The frequency of each respiratory and allergic symptom studied at each age and by identified groups is presented in Appendices S5 and S6. Overall, the frequency of wheezing, asthma medication, and eczema ranged between 12% and 28%, 22% and 24%, and 17% and 26% between the ages of 1 and 5.5 years, respectively. The frequency of allergic conjunctivitis was around 23% at ages of 3.5 and 5.5 years.

**FIGURE 2:**
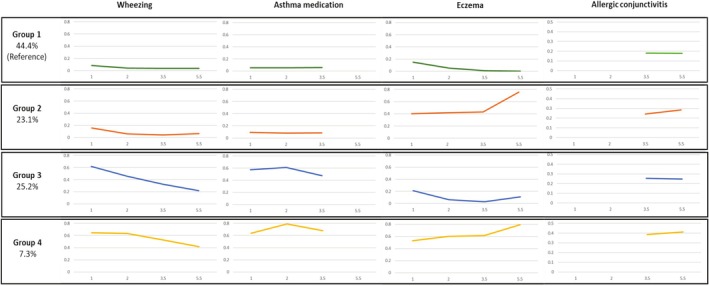
Multi‐trajectories of respiratory and allergic symptoms between 1 and 5.5 years (*N* = 10,524). Each graph presents the trajectory of the frequency of each ‘symptom’ as a function of the children's age. Groups included 4 ‘symptoms’: Wheezing, asthma medication, eczema, and allergic conjunctivitis. Group 1 = pauci‐symptomatic, Group 2 = persistent non‐respiratory allergic symptoms, Group 3 = transient early respiratory symptoms, Group 4 = persistent respiratory and allergic symptoms.

Four distinct respiratory and allergic symptoms multi‐trajectories (RespA‐MTG) between 1 and 5.5 years were identified in the 10,524 children included in the modelling (Figure 2).

Children belonging to the first group (G1, pauci‐symptomatic, 44.4% of children) had no or few respiratory and allergic symptoms. The frequency of wheezing decreased from 8.2% at 1 year to 4% at 5.5 years, while the frequency of asthma medication remained stable at about 4% at all ages. Children had a 15% frequency of eczema rash at age 1 year, which decreased to 5% at 2 years of age and zero at 5.5 years of age. The frequency of conjunctivitis was stable at about 18% at ages 3.5 and 5.5 years.

Children belonging to the second group (G2, persistent non‐respiratory allergic symptoms, 23.1%) had a frequency of eczema of about 42% between the ages of 1 and 3.5 years, increasing to 76% at 5.5 years, and a frequency of conjunctivitis of 23% and 29% between the ages of 3.5 and 5.5 years. Respiratory symptoms were uncommon, about 10%.

Children belonging to the third group (G3, transient early respiratory symptoms, 25.2%) had a frequency of wheezing of 63% at age 1 year, decreasing to 22% at 5.5 years, and a high frequency of asthma medication (> 50%) between 1 and 3.5 years. The frequency of eczema decreased from 21% at age 1 to 3% at age 3.5 years and increased slightly to 11% at age 5.5 years. The frequency of conjunctivitis remained stable at approximately 25% between the ages of 3.5 and 5.5 years.

Children belonging to the fourth group (G4, persistent respiratory and allergic symptoms, 7.3%) had high and stable wheezing frequency at 65% between the ages of 1 and 2 years, and then decreased slightly to 45% at age 5.5 years. The frequency of asthma medication was 64% at age 1, increased to 79% at age 2, and decreased slightly to 68% at age 3.5 years. The frequency of eczema was 53% at age 1 year and increased to 79% at age 5.5 years. Finally, the frequency of allergic conjunctivitis remained stable at approximately 40% between 3.5 and 5.5 years of age.

### 3.4 Children Assigned to Both Sleep Clusters and RespA‐MTG

A total of 9577 children had assignments to sleep clusters at age 1 year and 5.5 years and to RespA‐MTG groups (Appendix S1). Distributions of the children in the different clusters and multi‐trajectory groups were similar to the initial ones, with 18.2% and 13.2% of children in the cluster with poor sleep at age 1 year and 5.5 years, respectively. 23.8% of poor sleepers at age 1 year were still poor sleepers at age 5.5 years. Factors associated with each cluster at age 1 and 5.5 years are provided in Appendix S7. Between 1 and 5.5 years, 44.3%, 23.3%, 25.0%, and 7.4% of children were in pauci‐symptomatic, persistent non‐respiratory allergic symptoms, transient early respiratory symptoms, and persistent respiratory and allergic symptoms multi‐trajectory groups, respectively.

### 3.5 Associations Analysis

After adjustment for confounders, being a poor sleeper at age 1 (C2a) was associated with an increased odds of belonging to the persistent respiratory and allergic symptoms multi‐trajectory group between the ages of 1 and 5.5 years (G4) (adjusted odds ratio [95% confidence interval], 1.29 [1.05–1.59]) compared to the pauci‐symptomatic multi‐trajectory group (G1) (Table 2). Analysis in children without wheezing symptoms at age 2 months showed that being a poor sleeper at age 1 had an increased odds of belonging to the transient early respiratory symptoms multi‐trajectory group (G3) (1.18 [1.03–1.31]) and the persistent respiratory and allergic symptoms multi‐trajectory group (G4) (1.28 [1.03–1.60]) compared to the pauci‐symptomatic multi‐trajectory group (G1).

**TABLE 2:** Association between sleep clusters at age 1 year and respiratory and allergic multi‐trajectories between ages 1 and 5.5 (*N* = 9577). The pauci‐symptomatic group served as reference.

	Multi‐trajectory group		
	Persistent non‐respiratory allergic symptoms	Transient early respiratory symptoms	Persistent respiratory and allergic symptoms
Sleep clusters at 1yo	OR [95% CI]	OR [95% CI]	OR [95% CI]
Good sleepers	1.00 (reference)	1.00 (reference)	1.00 (reference)
In all children (*N* = 9577)			
Poor sleepers			
Unadjusted	1.06 [0.93–1.21]	1.10 [0.97–1.25]	1.25 [1.02–1.52]
Adjusted^a^	1.12 [0.98–1.29]	1.14 [0.99–1.31]	1.29 [1.05–1.60]
In children without wheezing at 2 months (*N* = 9054)			
Poor sleepers			
Unadjusted	1.05 [0.92–1.21]	1.13 [0.99–1.29]	1.24 [1.01–1.53]
Adjusted^a^	1.12 [0.98–1.29]	1.18 [1.03–1.36]	1.28 [1.03–1.60]

Abbreviations: CI, confidence interval; OR, odds ratio.

^a^ Adjusted for maternal education, household income, maternal age, mother birth place, maternal smoking during pregnancy, maternal pre‐pregnancy body mass index, parity, maternal depression symptoms during pregnancy, child sex, gestational age, breastfeeding duration, passive smoking exposure up to 1 yo, and child day‐care arrangement.

On the other hand, compared to the pauci‐symptomatic multi‐trajectory group (G1), belonging to the persistent respiratory and allergic symptoms multi‐trajectory group (G4) was associated with an increased odds of being a poor sleeper at age 5.5 years (C2b) (1.25 [1.02–1.54]). The effect size of the association was similar when analysis was carried out only among good sleepers at age 1 year (*N* = 7814), but did not reach the significance level after adjustment (1.24 [0.97–1.59]) (Table 3). Additionally, accounting for maternal history of asthma did not modify the relations (data not shown).

**TABLE 3:** Association between respiratory and allergic multi‐trajectories between 1 and 5.5 years and sleep clusters at 5.5 years. The good sleeper cluster at age 5.5 years served as reference.

	Poor sleepers at age 5.5 years			
	In all children (*N* = 9577)		In good sleepers at 1yo (*N* = 7814)	
	Unadjusted	Adjusted^a^	Unadjusted	Adjusted^a^
Respiratory and allergic multi‐trajectory groups	OR [95% CI]	OR [95% CI]	OR [95% CI]	OR [95% CI]
Pauci‐symptomatic	1.00	1.00	1.00	1.00
Persistent non‐respiratory allergic symptoms	1.09 [0.95–1.25]	1.11 [0.97–1.28]	1.09 [1.02–1.17]	1.11 [0.95–1.31]
Transient early respiratory symptoms	1.06 [0.92–1.21]	1.07 [0.93–1.22]	1.05 [0.98–1.13]	1.07 [0.91–1.26]
Persistent respiratory and allergic symptoms	1.24 [1.01–1.52]	1.25 [1.02–1.54]	1.23 [1.10–1.37]	1.24 [0.97–1.59]

Abbreviations: CI, confidence interval; OR, odds ratio.

^a^ Adjusted for maternal education, household income, maternal age, mother birth place, maternal smoking during pregnancy, parity, maternal depression symptoms during pregnancy, child sex, gestational age, breastfeeding duration, passive smoking exposure up to 1yo, and child day‐care arrangement.

## 4 Discussion

This study, using data from a birth cohort, provided new results regarding both sleep and respiratory and allergic health and their interrelations in preschoolers. Indeed, we identified (1) specific sleep clusters at age 1 and 5.5 years, (2) groups of trajectories of respiratory and allergic symptoms between 1 and 5.5 years, and (3) positive longitudinal associations between the cluster of children with poorer sleep at age 1 year and the multi‐trajectory groups of transient early respiratory symptoms and persistent respiratory and of allergic symptoms between 1 and 5.5 years, while a borderline positive association was observed between persistent respiratory and allergic symptoms between 1 and 5.5 years and sleep at 5.5 in good sleepers at 1 year.

We were able to identify clusters of children with different sleep characteristics at ages 1 and 5.5, in particular one cluster at each age with poor sleep—that is, with short sleep durations and frequent ‘frequent sleep onset difficulties’ and ‘frequent night waking’ representing 20%–17% of the children at age 1 and 5.5. A recent study, using latent class analysis, also identified two cross‐sectional clusters of sleep characteristics in children aged 6 months and 4 years, with one of the clusters presenting higher rates of night awakenings (Goncalves et al. 2023). Others reported longitudinal clusters of sleep characteristics considered one by one in children from birth cohorts, aged between 1 and 9 years old (Murcia et al. 2019; Plancoulaine et al. 2018; Reynaud et al. 2016). They all report about 20% of children with short sleep duration (Plancoulaine et al. 2018), frequent night awakenings (Murcia et al. 2019; Reynaud et al. 2016), and frequent sleep onset difficulties (Murcia et al. 2019; Reynaud et al. 2016) in line with our present results.

We identified 4 distinct groups of respiratory and allergic health trajectories, between 1 and 5.5 years old, with clinical significance. Several previous publications identified respiratory health trajectories in preschool children based on information collected by questionnaire. They identified 4 to 5 longitudinal phenotypes including or not multiple respiratory symptoms (Apel et al. 2019; Khan et al. 2023; Ranciere et al. 2013; Savenije et al. 2011) in 935–9517 children aged from 2 months to 5 years old. Phenotypes were then defined based on at least wheezing, allergic rhinitis/rhinitis symptoms, dermatitis rash/eczema, and night cough. None considered treatment. As we studied relations between respiratory and allergic health trajectories and sleep, we did not include night cough or sleep disturbances due to wheezing that were related to night waking, nor allergic rhinitis as it was collected only once. This may explain part of the differences observed. Another explanation could be the different sizes of the studied sample as the decision to select a phenotype or a group of multi‐trajectories needs altogether clinical significance, enough individuals per phenotype or group, parsimony, and fulfilled validation criteria.

We investigated longitudinal interrelations between sleep clusters at age 1 and 5.5 years and groups of trajectories of respiratory and allergic symptoms between the age of 1 and 5.5 years. The analysis showed that poor sleep in 1‐year‐old children is associated with poor respiratory and allergic status between 1 and 5.5 years of age, and that poor respiratory and allergic health between 1 and 5.5 years is associated with an increased risk of poor sleep in 5.5‐year‐old children. This last risk became borderline significant when sleep clusters at age 1 year were taken into account. Overall, these results suggest that sleep disturbances and respiratory and allergic troubles are closely linked from early life to preschool age. Longitudinal studies focusing on the association between sleep characteristics and subsequent respiratory and allergic symptoms or disease are very few. One study performed in 2398 Australian children from the Raine birth cohort showed that frequent night awakenings in the first 3 years of life were associated with the presence of non‐atopic asthma at age 6 and 14 years but not with atopic asthma (i.e., asthma + positive skin test) after accounting for the presence of wheeze before age 3 years (Kozyrskyj et al. 2009). A longitudinal study in 17,927 adults showed associations between each insomnia symptom (sleep onset difficulties, night waking, and nonrestorative sleep) and incident asthma 11 years later with a cumulative effect (Brumpton et al. 2017). These two studies, while exploring one facet of sleep in older populations, suggest that sleep disturbances can predict asthma symptoms. Similarly, in a recent study, the authors combined sleep characteristics using a score including chronotype, sleep duration, insomnia, snoring, and excessive daytime sleepiness in 455,405 adults aged 38–73 years from the UK Biobank dataset. They showed that asthma incidence after 10 years of follow‐up was higher in individuals with poorer sleep at baseline, independently of the genetic risk of asthma development. They calculated that healthy sleep patterns would theoretically reduce 19% of asthma cases in the population (Xiang et al. 2023). To our knowledge, longitudinal studies focusing on the reverse association, that is, respiratory and allergic symptoms or disease and sleep characteristics, focus mainly on sleep disruption due to night cough in asthmatic patients, thus signalling asthma severity. However, one very recent study using Mendelian randomisation in adult databases from Caucasian ancestry explored the causal relationships between insomnia, allergic disease (including asthma and/or hay fever and/or eczema), and asthma (moderate‐to‐severe, adult‐onset, and childhood‐onset asthma) (Li et al. 2022). They showed that in adults, insomnia was causally associated with an increased risk of allergic disease, asthma per se, and its phenotypes: moderate‐to‐severe asthma, and adult‐onset asthma. The reverse causal links were not supported (Li et al. 2022). This could be in line with our results showing significant adjusted associations between sleep disturbances at age 1 year and subsequent respiratory and allergic groups of trajectories (transient early respiratory symptoms multi‐trajectory group [G3] and persistent respiratory and allergic symptoms multi‐trajectory group [G4]) in children without wheezing symptoms at 2 months and borderline ones between those groups of trajectories and sleep disturbances at age 5.5 years in children without sleep disturbances at age 1 year. They did not find any causal link between childhood‐onset asthma and insomnia in adulthood, but data on childhood‐onset asthma was collected retrospectively while participants were 37–73 years old, potentially introducing under‐declaration and memory bias.

One of the main strengths of this study is the use of a prospective birth cohort design study collecting longitudinal data and allowing us to assess sleep, respiratory, and allergic health information with low memory bias. Another strength is the use of unsupervised clustering methods to identify both clusters of children with similar sleep patterns cross‐sectionally at two ages and developmental clusters of early respiratory and allergic health status over 5 years of time. Those two data‐driven methods identify latent groups with similar profiles accounting simultaneously for multiple traits or characteristics. All groups have clinical meaning and may help clinicians identify children to be particularly followed as ‘at risk’. The longitudinal unsupervised clustering allows for missing data; thus, we maximised the sample size to be analysed. Nevertheless, this study also presents some limitations. First, this is an observational study, and despite providing strong arguments, conclusions on causal inferences cannot be drawn. Second, children's sleep characteristics and respiratory and allergic symptoms were assessed by parental self‐reporting, possibly leading to under‐ or over‐self‐estimation. Parents usually overestimate child sleep duration based on bedtime and wake‐up time, as it reflects more time in bed than the exact sleep duration. The same is true for sleep onset difficulties, contrary to night waking, that might only be those that bother parents. Sleep characteristics might have been more accurately estimated by actigraphy, which has not been considered for cost and logistical reasons when the ELFE survey was designed in the 2010s (Charles et al. 2020). However, parental reports on their child's sleep characteristics are still broadly used in epidemiology and have been shown to be reliable compared to actigraphy (Iwasaki et al. 2010). Respiratory and allergic health symptoms might also be over‐or under‐estimated by parents. Biological and respiratory exploration markers would have allowed for a reliable diagnosis as recommended by respiratory societies (Louis et al. 2022) but have not been performed in this large epidemiological cohort. Third, we did not include in the multi‐trajectories modelling information on allergic rhinitis, collected only at age 5.5 years, as we included respiratory and allergic symptoms only when available at least at two time points between 1 and 5.5 years old. Finally, the studied population is specific, with high socioeconomic status, high maternal educational level, and healthy lifestyle, limiting the generalisation of our results to the French population. However, as sleep disturbances and poorer respiratory and allergic symptoms are reported more frequently in less favourable living contexts (Caffrey Osvald et al. 2022; Sosso and Khoury 2021), the associations reported here could be reinforced among more disfavoured and vulnerable children. This will need to be further studied.

## 5 Conclusion

This study provides arguments for the prediction of respiratory and allergic health by early sleep disturbances as early as infancy. Sleep could be a potential target for interventions to alleviate allergic and respiratory symptoms in preschoolers. This study, however, suggests that a good control of respiratory and allergic symptoms could benefit subsequent sleep disturbances.

## Author Contributions

**Daniele Saade:** writing – original draft, data curation, formal analysis, visualization, writing – review and editing. **Rosalie Delvert:** validation, writing – review and editing. **Chantal Raherison‐Semjen:** validation, writing – review and editing, investigation. **Orianne Dumas:** writing – review and editing, validation. **Mohammed Sedki:** methodology, writing – review and editing. **Marie‐Noëlle Dufourg:** investigation, writing – review and editing. **Blandine de Lauzon‐Guillain:** validation, writing – review and editing. **Bénédicte Leynaert:** validation, writing – review and editing, investigation. **Rachel Nadif:** funding acquisition, validation, writing – review and editing. **Annabelle Bédard:** validation, writing – review and editing. **Sabine Plancoulaine:** conceptualization, funding acquisition, data curation, formal analysis, methodology, validation, writing – review and editing, project administration, supervision.

## Ethics Statement

The study protocol and the collection of stools were approved by the Committee for the Protection of People Participating in Biomedical Research (Comité de Protection des Personnes [CPP]), the national advisory committee on information processing in health research (Comité Consultatif sur le Traitement de l'Information en matière de Recherche dans le domaine de la Santé [CCTIRS]), and the French National Data Protection Authority (Commission Nationale de l'Informatique et des Libertes [CNIL]). Recruitment and data collection involved families that had received information and provided consent to participate.

## Conflicts of Interest

The authors declare no conflicts of interest.

## Supporting information


**Appendix S1:** Supporting Information.

## Data Availability

The data underlying this article cannot be shared publicly due to the privacy of individuals who participated in the study. The data will be shared on reasonable request to the cohort scientific committee.
