# Supplementary material for: Sleep and Trajectories of Respiratory and Allergic Symptoms Between 1 and 5.5 Years of Age in the Elfe Birth Cohort
Source: J Sleep Res. 2025 Sep 16;35(2):e70208. doi: 10.1111/jsr.70208 (PMC13003267; doi:10.1111/jsr.70208)
Supplement: Supplementary file 1 — Appendix S1: Supporting Information. [file JSR-35-e70208-s001.docx]

**Appendices**

1. Flow chart
2. Questions and collection dates
3. Directed acyclic graphs at age 1 and 5.5 years
4. Maternal and children characteristics of the study excluded and included population
5. Frequency of respiratory and allergic symptoms in the last 12 months, according to child age, from 1 to 5.5 years (N=10,524)
6. Frequency of respiratory and allergic symptoms in the last 12 months, according to multi-trajectory groups (N=9,577)
7. Factors associated with each cluster at age 1 and 5.5 years (N=9,577)
8. Factors associated with respiratory and allergic symptoms

**Appendix 1. Flow chart**

**Appendix 2. Questions and collection dates**

| **Questions** | **Possible answers** | **Collection points** | | | | | | | | | | |  |
| --- | --- | --- | --- | --- | --- | --- | --- | --- | --- | --- | --- | --- | --- |
|  |  | 2 months | | 1 year | | 2 years | | 3.5 years | | 5.5 years | |  |  |
| **Sleep** |  |  | |  | |  | |  | |  | |  |  |
| What is your child’s total night sleep duration | Duration (hrs & min) |  | | x | |  | |  | |  | |  |  |
| What is your child’s total daytime sleep duration including naps | Duration (hrs & min) |  | | x | |  | |  | |  | |  |  |
| Usually, at what time does your child go to bed? | Clock time (hrs & min) (weekdays & weekend days) |  | |  | |  | |  | | x | |  |  |
| Usually, at what time does your child wake up? | Clock time (hrs & min) (weekdays & weekend days) |  | |  | |  | |  | | x | |  |  |
| How many nights did your child wake up during the night this week? | Never, 1 or 2 nights, 3 to 6 nights and always |  | | x | |  | |  | | x | |  |  |
| When you put your child in the bed, does he/she have difficulties falling asleep? For example, he/she calls or cries for a long time over 30 minutes? | Never, sometimes and often |  | | x | |  | |  | |  | |  |  |
|  | Never, sometimes, often and always |  | |  | |  | |  | | x | |  |  |
|  |  |  | |  | |  | |  | |  | |  |  |
| **Respiratory and allergic symptoms** |  |  | |  | |  | |  | |  | |  |  |
| Has your child had any wheezing in the chest? | Yes/no | x | |  | |  | |  | |  | |  |  |
| Has your child had wheezing in the chest in the last 12 months? | Yes/no |  | | x | | x | | x | | x | |  |  |
| Has your child ever received inhaled medicines at home or in hospital to improve breathing at any time in the past 12 months? | Yes/no |  | | x | |  | |  | |  | |  |  |
| If yes, specify if it is an inhaled bronchodilator and/or an inhaled corticosteroid |  |  | |  | |  | |  | |  | |  |  |
| Bronchodilators (Bricanyl (terbutaline), Formoterol (formoterol fumarate), Ventolin (salbutamol), Seretide (salmeterol + fluticasone)) | | |  | | x | |  | |  | |  | | |
| Corticosteroids (Beclospin (beclometasone), Becotide (beclometasone), Budesonide (budesonide), Pulmicort (budesonide), Flixotide (fluticasone propionate), Seretide (salmeterol + fluticasone)) | |  | | x | |  | |  | |  | |  |  |
| Has your child ever received any inhaled medications at home or in hospital to improve breathing such as Ventolin, Bricanyl, Pulmicort, Flixotide, Becotide, at any time in the last 12 months? | Yes/no |  | |  | | x | |  | |  | | | |
| In the past 12 months, has your child ever received Ventolin or equivalent? | Yes/no |  | |  | |  | | x | |  | | | |
| In the past 12 months, has your child ever received inhaled or nebulized corticosteroids such as Becotide, Flixotide? | Yes/no |  | |  | |  | | x | |  | | | |
| Has your child ever had a rash on the skin (red pimple patches, etc.) that itches (like to scratch) and that appears and disappears intermittently | Yes/no |  | | x | |  | |  | |  | | | |
| Has your child ever had eczema flare-ups in the last 12 months? | Yes/no |  | |  | | x | | x | |  | | | |
| Has your child ever had eczema? | Yes/no |  | |  | |  | |  | | x | | | |
| Has your child ever had an eye allergy, allergic conjunctivitis? | Yes/no |  | |  | |  | | x | | x | | | |

**Appendix 3. Directed acyclic graphs (DAG) at age 1 and 5.5 years.**

**DAG 1. Sleep at 1 year old (yo) as exposure and respiratory and allergic multi-trajectories as outcome**

- **Graph:**

**
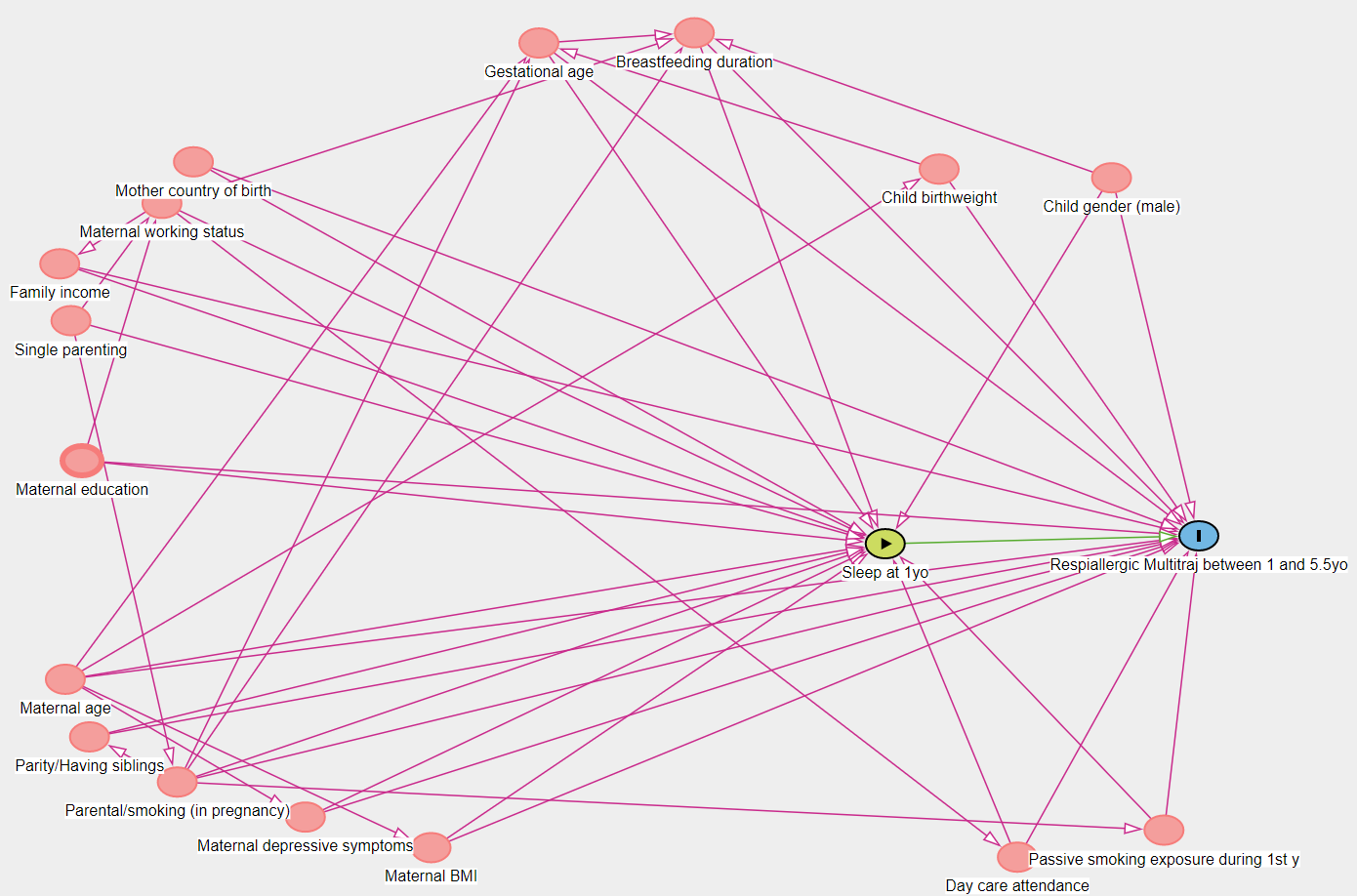
**

- **Minimal sufficient adjustment sets for estimating the total effect:**

Breastfeeding duration, Child gender (male), Day care attendance, Family income, Gestational age, Maternal BMI, Maternal age, Maternal depressive symptoms, Maternal education, Mother country of birth, Parental/smoking (in pregnancy), Parity/Having siblings, Passive smoking exposure during 1st year

- **Code:**

dag {

bb="-6.366,-4.912,5.856,5.581"

"Breastfeeding duration" [pos="-0.266,-4.496"]

"Child birthweight" [pos="1.873,-2.908"]

"Child gender (male)" [pos="3.379,-2.807"]

"Day care attendance" [pos="2.556,5.106"]

"Family income" [pos="-5.810,-1.805"]

"Gestational age" [pos="-1.624,-4.377"]

"Maternal BMI" [pos="-2.566,4.995"]

"Maternal age" [pos="-5.761,3.034"]

"Maternal depressive symptoms" [pos="-3.664,4.639"]

"Maternal education" [pos="-5.782,0.564"]

"Maternal working status" [pos="-4.917,-2.509"]

"Mother country of birth" [pos="-4.642,-2.993"]

"Parental/smoking (in pregnancy)" [pos="-4.783,4.231"]

"Parity/Having siblings" [pos="-5.550,3.705"]

"Passive smoking exposure during 1st y " [pos="3.837,4.791"]

"Respiallergic Multitraj between 1 and 5.5yo" [outcome,pos="4.899,1.489"]

"Single parenting" [pos="-5.712,-1.143"]

"Sleep at 1yo" [exposure,pos="1.402,1.455"]

"Breastfeeding duration" -> "Respiallergic Multitraj between 1 and 5.5yo"

"Breastfeeding duration" -> "Sleep at 1yo"

"Child birthweight" -> "Gestational age"

"Child birthweight" -> "Respiallergic Multitraj between 1 and 5.5yo"

"Child gender (male)" -> "Breastfeeding duration"

"Child gender (male)" -> "Respiallergic Multitraj between 1 and 5.5yo"

"Child gender (male)" -> "Sleep at 1yo"

"Day care attendance" -> "Respiallergic Multitraj between 1 and 5.5yo"

"Day care attendance" -> "Sleep at 1yo"

"Family income" -> "Respiallergic Multitraj between 1 and 5.5yo"

"Family income" -> "Sleep at 1yo"

"Gestational age" -> "Breastfeeding duration"

"Gestational age" -> "Respiallergic Multitraj between 1 and 5.5yo"

"Gestational age" -> "Sleep at 1yo"

"Maternal BMI" -> "Respiallergic Multitraj between 1 and 5.5yo"

"Maternal BMI" -> "Sleep at 1yo"

"Maternal age" -> "Child birthweight"

"Maternal age" -> "Gestational age"

"Maternal age" -> "Maternal BMI"

"Maternal age" -> "Maternal depressive symptoms"

"Maternal age" -> "Respiallergic Multitraj between 1 and 5.5yo"

"Maternal age" -> "Sleep at 1yo" 2

"Maternal depressive symptoms" -> "Respiallergic Multitraj between 1 and 5.5yo"

"Maternal depressive symptoms" -> "Sleep at 1yo"

"Maternal education" -> "Maternal working status"

"Maternal education" -> "Respiallergic Multitraj between 1 and 5.5yo"

"Maternal education" -> "Sleep at 1yo"

"Maternal working status" -> "Breastfeeding duration"

"Maternal working status" -> "Day care attendance"

"Maternal working status" -> "Family income"

"Maternal working status" -> "Sleep at 1yo"

"Mother country of birth" -> "Respiallergic Multitraj between 1 and 5.5yo"

"Mother country of birth" -> "Sleep at 1yo"

"Parental/smoking (in pregnancy)" -> "Breastfeeding duration"

"Parental/smoking (in pregnancy)" -> "Gestational age"

"Parental/smoking (in pregnancy)" -> "Parity/Having siblings"

"Parental/smoking (in pregnancy)" -> "Passive smoking exposure during 1st y "

"Parental/smoking (in pregnancy)" -> "Respiallergic Multitraj between 1 and 5.5yo"

"Parental/smoking (in pregnancy)" -> "Sleep at 1yo"

"Parity/Having siblings" -> "Respiallergic Multitraj between 1 and 5.5yo"

"Parity/Having siblings" -> "Sleep at 1yo"

"Passive smoking exposure during 1st y " -> "Respiallergic Multitraj between 1 and 5.5yo"

"Passive smoking exposure during 1st y " -> "Sleep at 1yo"

"Single parenting" -> "Maternal working status"

"Single parenting" -> "Parental/smoking (in pregnancy)"

"Single parenting" -> "Sleep at 1yo"

"Sleep at 1yo" -> "Respiallergic Multitraj between 1 and 5.5yo"

}

**DAG 2. Respiratory and allergic multi-trajectories as exposure and sleep at 5.5yo as outcome**

- **Graph:**

**
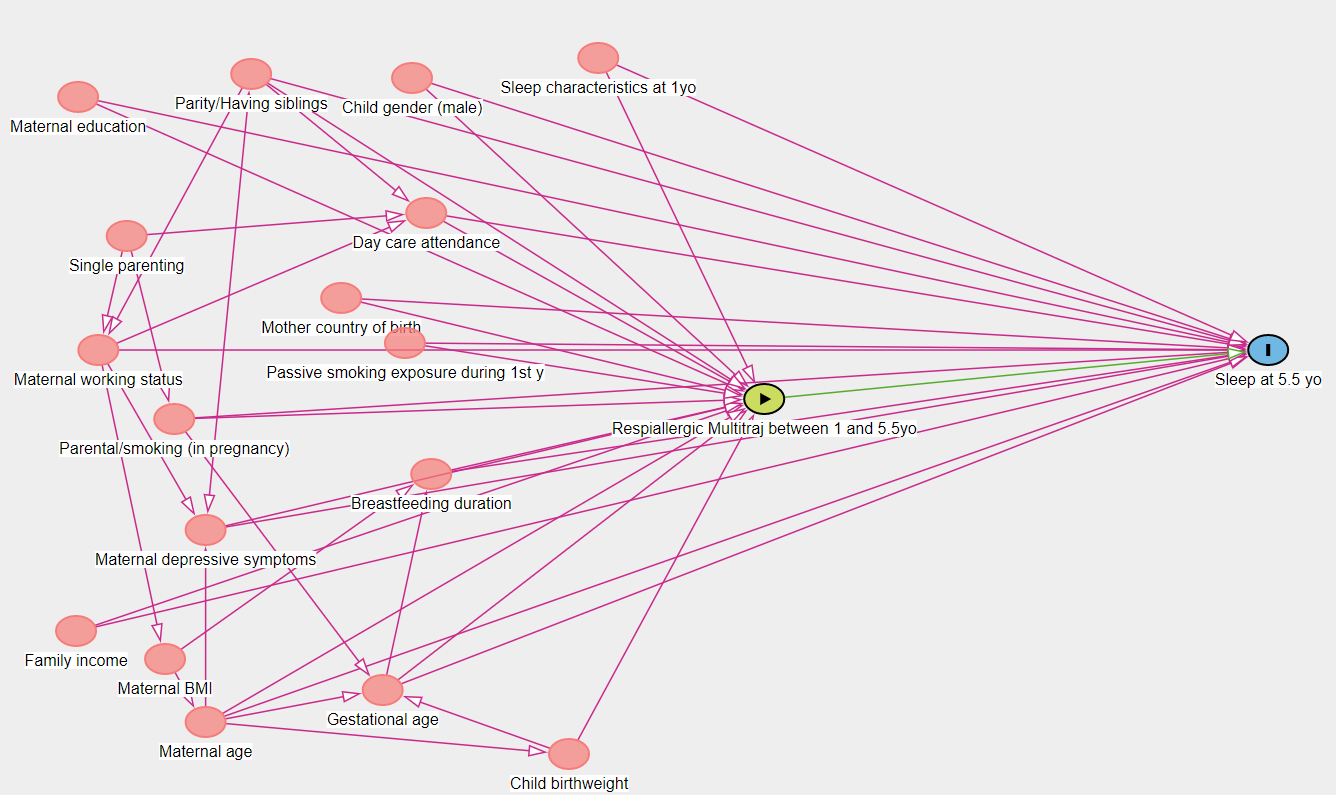
**

- **Minimal sufficient adjustment sets for estimating the total effect:**

Breastfeeding duration, Child gender (male), Day care attendance, Family income, Gestational age, Maternal age, Maternal depressive symptoms, Maternal education, Mother country of birth, Parental/smoking (in pregnancy), Parity/Having siblings, Passive smoking exposure during 1st y , sleep characteristics at 1yo.

- **Code:**

dag {

bb="-7.449,-6.117,9.128,5.602"

"Breastfeeding duration" [pos="-2.310,0.282"]

"Child birthweight" [pos="-0.716,3.837"]

"Child gender (male)" [pos="-2.532,-4.746"]

"Day care attendance" [pos="-2.368,-3.032"]

"Family income" [pos="-6.419,2.275"]

"Gestational age" [pos="-2.872,3.025"]

"Maternal BMI" [pos="-5.389,2.631"]

"Maternal age" [pos="-4.920,3.431"]

"Maternal depressive symptoms" [pos="-4.920,0.993"]

"Maternal education" [pos="-6.395,-4.505"]

"Maternal working status" [pos="-6.161,-1.292"]

"Mother country of birth" [pos="-3.352,-1.953"]

"Parental/smoking (in pregnancy)" [pos="-5.283,-0.416"]

"Parity/Having siblings" [pos="-4.393,-4.797"]

"Passive smoking exposure during 1st y " [pos="-2.614,-1.381"]

"Respiallergic Multitraj between 1 and 5.5yo" [exposure,pos="1.542,-0.670"]

"Single parenting" [pos="-5.833,-2.740"]

"Sleep at 5.5 yo" [outcome,pos="7.372,-1.292"]

"Sleep characteristics at 1yo" [pos="-0.378,-5.000"]

"Breastfeeding duration" -> "Respiallergic Multitraj between 1 and 5.5yo"

"Breastfeeding duration" -> "Sleep at 5.5 yo"

"Child birthweight" -> "Gestational age"

"Child birthweight" -> "Respiallergic Multitraj between 1 and 5.5yo"

"Child gender (male)" -> "Respiallergic Multitraj between 1 and 5.5yo"

"Child gender (male)" -> "Sleep at 5.5 yo"

"Day care attendance" -> "Respiallergic Multitraj between 1 and 5.5yo"

"Day care attendance" -> "Sleep at 5.5 yo"

"Family income" -> "Respiallergic Multitraj between 1 and 5.5yo"

"Family income" -> "Sleep at 5.5 yo"

"Gestational age" -> "Breastfeeding duration"

"Gestational age" -> "Respiallergic Multitraj between 1 and 5.5yo"

"Gestational age" -> "Sleep at 5.5 yo"

"Maternal BMI" -> "Breastfeeding duration"

"Maternal BMI" -> "Maternal age"

"Maternal age" -> "Child birthweight"

"Maternal age" -> "Gestational age"

"Maternal age" -> "Maternal depressive symptoms"

"Maternal age" -> "Respiallergic Multitraj between 1 and 5.5yo" 4

"Maternal age" -> "Sleep at 5.5 yo"

"Maternal depressive symptoms" -> "Respiallergic Multitraj between 1 and 5.5yo"

"Maternal depressive symptoms" -> "Sleep at 5.5 yo"

"Maternal depressive symptoms" <-> "Parity/Having siblings"

"Maternal education" -> "Respiallergic Multitraj between 1 and 5.5yo"

"Maternal education" -> "Sleep at 5.5 yo"

"Maternal working status" -> "Day care attendance"

"Maternal working status" -> "Maternal BMI"

"Maternal working status" -> "Maternal depressive symptoms"

"Maternal working status" -> "Sleep at 5.5 yo"

"Mother country of birth" -> "Respiallergic Multitraj between 1 and 5.5yo"

"Mother country of birth" -> "Sleep at 5.5 yo"

"Parental/smoking (in pregnancy)" -> "Gestational age"

"Parental/smoking (in pregnancy)" -> "Respiallergic Multitraj between 1 and 5.5yo"

"Parental/smoking (in pregnancy)" -> "Sleep at 5.5 yo"

"Parity/Having siblings" -> "Day care attendance"

"Parity/Having siblings" -> "Maternal working status"

"Parity/Having siblings" -> "Respiallergic Multitraj between 1 and 5.5yo"

"Parity/Having siblings" -> "Sleep at 5.5 yo"

"Passive smoking exposure during 1st y " -> "Respiallergic Multitraj between 1 and 5.5yo"

"Passive smoking exposure during 1st y " -> "Sleep at 5.5 yo"

"Respiallergic Multitraj between 1 and 5.5yo" -> "Sleep at 5.5 yo"

"Single parenting" -> "Day care attendance"

"Single parenting" -> "Maternal working status"

"Single parenting" -> "Parental/smoking (in pregnancy)"

"Sleep characteristics at 1yo" -> "Respiallergic Multitraj between 1 and 5.5yo"

"Sleep characteristics at 1yo" -> "Sleep at 5.5 yo"

}

**Appendix 4: Maternal and children characteristics of the study excluded and included population**

|  |  | **Included** | **Excluded** | ***p*-value** |
| --- | --- | --- | --- | --- |
|  |  | **N=9,577** | **N=7,046** |  |
|  |  | % (n) | % (n) |  |
|  |  | or mean (SD^†^) | or mean (SD^†^) |  |
| **Socio-demographic characteristics** | | |  |  |
| Maternal educational level | |  |  | <.0001 |
|  | < High school | 30.0 (2869) | 61.4 (3557) |  |
|  | High school | 24.1 (2312) | 17.7 (1023) |  |
|  | > High school | 45.9 (4396) | 21.0 (1214) |  |
| Household income (€/month/CU^‡^) | | |  | <.0001 |
|  | ≤ 1000 | 12.5 (1154) | 33.9 (1684) |  |
|  | ]1000 - 1385] | 18.2 (1676) | 22.7 (1128) |  |
|  | ]1385-1662] | 18.1 (1665) | 14.6 (724) |  |
|  | ]1662 - 2078] | 26.4 (2436) | 16.7 (833) |  |
|  | > 2078 | 24.8 (2285) | 12.2 (605) |  |
| **Maternal characteristics** | | |  |  |
| Age (years) | | 31.4 (4.5) | 29.5 (5.5) | <.0001 |
| Born abroad | | 8.8 (844) | 19.3 (1352) | <.0001 |
| Smoking during pregnancy | | 15.7 (1496) | 25.9 (1795) | <.0001 |
| BMI^$^ before pregnancy (kg/m^2^) | | 23.3 (4.5) | 23.8 (5.1) | <.0001 |
| Multiparous | | 65.6 (6264) | 67.9 (4761) | 0.002 |
| Depressive symptoms during pregnancy | | 11.8 (1119) | 12.8 (893) | 0.04 |
| **Children characteristics and early factors at 1-year-old** | | | | |
| Sex (boy) | | 50.9 (4879) | 51.6 (3637) | 0.39 |
| Gestational age (weeks) | | 39.5 (1.1) | 39.4 (1.1) | <.0001 |
| Breastfeeding duration (months) | | 4.1 (5.7) | 1.9 (4.2) | <.0001 |
| Passive smoking exposure up to 1-year-old | | 3.5 (328) | 8.2 (260) | <.0001 |
| Main day-care arrangement | |  |  | <.0001 |
|  | Cared by family members | 35.7 (3392) | 57.1 (2051) |  |
|  | Collective care | 17.6 (1668) | 12.5 (448) |  |
|  | Cared by employed person | 46.7 (4439) | 30.4 (1093) |  |

^†^SD, Standard deviation; ^‡^ CU, Consumption unit; BMI, Body mass index.

**Appendix 5: Frequency of respiratory and allergic symptoms in the last 12 months, according to the child age, from 1 to 5.5 years (N=10,524)**

|  | **No** | | **Yes** | |
| --- | --- | --- | --- | --- |
|  | **n** | **%** | **n** | **%** |
| **At 1 year** | | | | |
| Wheezing | 7441 | 72.1 | 2874 | 27.9 |
| Asthma medication^†^ | 7856 | 76.2 | 2459 | 23.8 |
| Eczema | 7661 | 74.3 | 2652 | 25.7 |
| **At 2 years** | | | | |
| Wheezing | 8260 | 80.3 | 2023 | 19. 7 |
| Asthma medication^†^ | 7650 | 74.4 | 2633 | 25.6 |
| Eczema | 8322 | 80.9 | 1961 | 19.1 |
| **At 3.5 years** | | | | |
| Wheezing | 8967 | 85.2 | 1557 | 14.8 |
| Asthma medication^†^ | 8249 | 78.4 | 2275 | 21.6 |
| Eczema | 8750 | 83.1 | 1774 | 16.9 |
| Allergic conjunctivitis | 8101 | 77.0 | 2423 | 23.0 |
| **At 5.5 years** | | | | |
| Wheezing | 9269 | 88.1 | 1255 | 11.9 |
| Eczema | 7555 | 71.9 | 2949 | 28.1 |
| Allergic conjunctivitis | 7996 | 76.0 | 2528 | 24.0 |

^†^ Asthma medication defined as use of any inhaled bronchodilator and/or inhaled corticosteroid

**Appendix 6: Frequency of respiratory and allergic symptoms in the last 12 months, according to multi-trajectory groups (N=9,577)**

|  | Wheezing | | Asthma medication | Eczema | | | Conjunctivitis | | |  |
| --- | --- | --- | --- | --- | --- | --- | --- | --- | --- | --- |
| Group and age | Frequency (%) | | Frequency (%) | Frequency (%) | | | Frequency (%) | | |  |
| Pauci-symptomatic | | | | |  | | | |  | |
| 1 | 8.1 | | 5.2 | 14.7 | | |  | | |  |
| 2 | 4.3 | | 5.2 | 5.3 | | |  | | |  |
| 3.5 | 3.6 | | 5.5 | 1.2 | | | 18.0 | | |  |
| 5.5 | 3.78 | |  | 0.2 | | | 17.7 | | |  |
| Persistent non-respiratory allergic symptoms | | | | |  | | | |  | |
| 1 | 15.8 | | 9.5 | 40.2 | | |  | | |  |
| 2 | 6.1 | | 8.0 | 41.8 | | |  | | |  |
| 3.5 | 4.1 | | 8.5 | 42.9 | | | 24.2 | | |  |
| 5.5 | 6.5 | |  | 75.8 | | | 28.6 | | |  |
| Transient early respiratory symptoms | | | | |  | | | |  | |
| 1 | 62.1 | | 57.5 | 21.2 | | |  | | |  |
| 2 | 45.7 | | 61.2 | 6.3 | | |  | | |  |
| 3.5 | 32.6 | | 47.6 | 2.8 | | | 25.5 | | |  |
| 5.5 | 21.7 | |  | 11.3 | | | 24.8 | | |  |
| Persistent respiratory and allergic symptoms | | | | | | | |  | | |
| 1 | 64.6 | 64.0 | | 52.8 | |  | | | |  |
| 2 | 63. 5 | 79.1 | | 60.0 | |  | | | |  |
| 3.5 | 52.7 | 68.2 | | 61.6 | | 38.6 | | | |  |
| 5.5 | 41.9 |  | | 79.2 | | 41.2 | | | |  |

**Appendix 7: Factors associated with each cluster at age 1 and 5.5 years (N=9,577)**

|  |  |  |  |  |  |  |  |  |
| --- | --- | --- | --- | --- | --- | --- | --- | --- |
|  |  | **Age 1 year** | | |  | **Age 5.5 years** | | |
|  |  | Good sleepers | Poor sleepers | p-val |  | Good sleepers | Poor  sleepers | p-val |
|  |  | % (n) or mean (SD^†^) | % (n) or mean (SD^†^) |  |  | % (n) or mean (SD^†^) | % (n) or mean (SD^†^) |  |
| **Socio-demographic characteristics** | |  |  |  |  |  |  |  |
| Maternal educational level | |  |  |  |  |  |  |  |
|  | < High school | 28.4 (1700) | 36.7 (2193) |  |  | 28.9 (1730) | 35.1 (2096) |  |
|  | High school | 24.6 (1471) | 22.1 (1319) | <10^-4^ |  | 24.7 (1479) | 21.1 (1262) | <10^-4^ |
|  | > High school | 47.0 (2806) | 41.2 (2465) | <10^-4^ |  | 46.3 (2768) | 43.8 (2618) | 0.0001 |
| Household income (€/month/CU^‡^) | |  |  |  |  |  |  |  |
|  | ≤ 1000 | 11.2 (671) | 22.4 (1338) |  |  | 12.7 (759) | 16.2 (968) |  |
|  | ]1000 – 1385] | 17.8 (1065) | 20.2 (1208) | <10^-4^ |  | 18.2 (1086) | 18.5 (1104) | 0.02 |
|  | ]1385-1662] | 18.2 (1086) | 16.4 (979) | <10^-4^ |  | 18.0 (1077) | 17.0 (1017) | 0.002 |
|  | ]1662 – 2078] | 27.1 (1623) | 20.8 (1241) | <10^-4^ |  | 26.4 (1581) | 23.6 (1410) | 0.0001 |
|  | > 2078 | 25.6 (1532) | 20.3 (1211) | <10^-4^ |  | 24.6 (1472) | 24.7 (1478) | 0.007 |
| **Maternal characteristics** | |  |  |  |  |  |  |  |
| Age (years) | | 31.5 (4.5) | 31.9 (4.9) | 0.001 |  | 31.6 (4.5) | 31.7 (4.6) | 0.22 |
| Born abroad | | 6.7 (403) | 18.0 (1075) | <10^-4^ |  | 8.3 (496) | 11.4 (905) | 0.0001 |
| Smoking during pregnancy | | 15.7 (939) | 15.8 (943) | 0.94 |  | 14.9 (893) | 19.7 (1177) | <10^-4^ |
| BMI^$^ before pregnancy (kg/m^2^) | | 23.2 (4.46) | 23.6 (4.88) | 0.0003 |  | 23.3 (4.5) | 23.2 (4.6) | 0.34 |
| Multiparous | | 65.1 (3889) | 67.8 (4054) | 0.03 |  | 65.8 (3931) | 64.6 (3860) | 0.37 |
| Depressive symptoms during pregnancy | | 11.1 (666) | 14.5 (868) | 0.0001 |  | 11.2 (671) | 14.4 (861) | 0.0004 |
| **Children characteristics and early factors at 1-year-old** | |  |  |  |  |  |  |  |
| Sex (boy) | | 50.3 (3008) | 53.7 (3211) | 0.01 |  | 50.8 (3035) | 48.2 (2881) | 0.46 |
| Gestational age (weeks) | | 39.5 (1.1) | 39.4 (1.1) | 0.003 |  | 39.5 (1.1) | 39.4 (1.2) | 0.23 |
| Breastfeeding duration (months) | | 3.6 (5.0) | 6.5 (7.9) | <10^-4^ |  | 4.1 (5.6) | 4.6 (6.4) | 0.0006 |
| Passive smoking exposure | | 3.3 (197) | 4.5 (271) | 0.01 |  | 3.4 (203) | 4.2 (252) | 0.10 |
| Main day-care arrangement | |  |  |  |  |  |  |  |
|  | Cared by family members | 32.6 (1948) | 49.2 (2942) |  |  | 34.8 (2081) | 39.9 (2383) |  |
|  | Collective care | 17.3 (1033) | 18.8 (1124) | <10^-4^ |  | 17.5 (1044) | 18.0 (1078) | 0.19 |
|  | Cared by employed person | 50.1 (2995) | 32.0 (1911) | <10^-4^ |  | 47.7 (2852) | 42.1 (2516) | <10^-4^ |

^†^SD, Standard deviation; ^‡^ CU, Consumption unit; BMI, Body mass index.

**Appendix 8: Factors associated with respiratory and allergic symptoms (N=9,577)**

|  |  | **Respiratory and allergic multi-trajectories between ages 1 and 5.5** | | | | | |  |
| --- | --- | --- | --- | --- | --- | --- | --- | --- |
|  |  | Pauci-symptomatic | Persistent non-respiratory allergic symptoms | | Transient early respiratory symptoms | Persistent respiratory and allergic symptoms | p-val |  |
|  |  |  |  |  |  |  |  |  |
|  |  | % (n) or mean (SD^†^) | % (n) or mean (SD^†^) | | % (n) or mean (SD^†^) | % (n) or mean (SD^†^) |  |  |
| **Socio-demographic characteristics** | | | | |  |  |  |  |
| Maternal educational level | | | |  |  |  | <10^-4^ |  |
|  | < High school | 31 (1317) | 28 (626) | | 29.9 (715) | 29.9 (211) |  |  |
|  | High school | 22.8 (970) | 24.7 (552) | | 25.8 (617) | 24.5 (173) |  |  |
|  | > High school | 46.2 (1961) | 47.2 (1054) | | 44.3 (1060) | 45.5 (321) |  |  |
| Household income (€/month/CU^‡^) | | | | |  |  | <10^-4^ |  |
|  | ≤ 1000 | 13.6 (576) | 12.7 (282) | | 13.6 (326) | 12.3 (87) |  |  |
|  | ]1000 – 1385] | 18.6 (791) | 17.9 (399) | | 18.2 (435) | 17.5 (124) |  |  |
|  | ]1385-1662] | 17.1 (727) | 17.2 (383) | | 18.7 (448) | 21.5 (152) |  |  |
|  | ]1662 – 2078] | 26.1 (1108) | 26.1 (583) | | 26.1 (623) | 24.5 (172) |  |  |
|  | > 2078 | 24.6 (1045) | 26.2 (584) | | 23.4 (560) | 24.1 (170) |  |  |
| **Maternal characteristics** | | | | |  |  |  |  |
| Age (years) | | 31.6 (4.6) | 31.5 (4.5) | | 31.7 (4.4) | 31.2 (4.3) | <10^-4^ |  |
| Born abroad | | 10.1 (431) | 7.9 (176) | | 7.5 (179) | 8.2 (58) | <10^-4^ |  |
| Smoking during pregnancy | | 15 (639) | 13.8 (308) | | 17.9 (429) | 18.5 (130) | <10^-4^ |  |
| BMI^$^ before pregnancy (kg/m^2^) | | 23.2 (4.4) | 23.1 (4.4) | | 23.5 (4.7) | 23.5 (4.7) | <10^-4^ |  |
| Multiparous | | 63.6 (2702) | 63.4 (1415) | | 70 (1675) | 69.2 (488) | <10^-4^ |  |
| Depressive symptoms during pregnancy | | 10.4 (443) | 11.8 (263) | | 13.3 (318) | 14.4 (101) | <10^-4^ |  |
| **Children characteristics and early factors at 1-year-old** | | | | | | |  |  |
| Sex (boy) | | 46.3 (1966) | 48.2 (1075) | | 57.9 (1385) | 64.3 (453) | <10^-4^ |  |
| Gestational age (weeks) | | 39.5 (1.1) | 39.5 (1.1) | | 39.4 (1.1) | 39.4 (1.2) | <10^-4^ |  |
| Breastfeeding duration (months) | | 4.3 (5.9) | 4.2 (5.6) | | 3.9 (5.4) | 4 (5.7) | <10^-4^ |  |
| Passive smoking exposure | | 3.3 (141) | 3.8 (84) | | 3.7 (89) | 3.4 (24) | 0.13 |  |
| Main day-care arrangement | | | |  |  |  | <10^-4^ |  |
|  | Cared by family members | 38.2 (1622) | 33.4 (746) | | 33.8 (808) | 33.8 (238) |  |  |
|  | Collective care | 14.8 (631) | 18.2 (406) | | 21.1 (504) | 20.1 (142) |  |  |
|  | Cared by employed person | 47 (1996) | 48.4 (1080) | | 45.1 (1080) | 46.1 (325) |  |  |

^†^SD, Standard deviation; ^‡^ CU, Consumption unit; BMI, Body mass index.
